# Inbred Mice Again at Stake: How the Cognitive Profile of the Wild-Type Mouse Background Discloses Pathogenic Effects of APP Mutations

**DOI:** 10.3389/fnbeh.2022.868473

**Published:** 2022-06-23

**Authors:** Martine Ammassari-Teule

**Affiliations:** ^1^Laboratory of Psychobiology, Department of Experimental Neuroscience, Santa Lucia Foundation, Rome, Italy; ^2^National Research Council, Institute of Biochemistry and Cell Biology, Rome, Italy

**Keywords:** inbred mice, cognitive challenges, genetic background, AD-related mutations, cognitive profile

## Abstract

Increasing efforts have been made in the last decades to increase the face validity of Alzheimer's disease (AD) mouse models. Main advancements have consisted in generating AD mutations closer to those identified in humans, enhancing genetic diversity of wild-type backgrounds, and choosing protocols much apt to reveal AD-like cognitive dysfunctions. Nevertheless, two aspects remain less considered: the cognitive specialization of inbred strains used as recipient backgrounds of mutations and the heuristic importance of studying destabilization of memory circuits in pre-symptomatic mice facing cognitive challenges. This article underscores the relevance of these behavioral/experimental aspects by reviewing data which show that (i) inbred mice differ in their innate predisposition to rely on episodic vs. procedural memory, which implicates differential sensitivity to mutations aimed at disrupting temporal lobe-dependent memory, and that (ii) investigating training-driven neural alterations in asymptomatic mutants unveils early synaptic damage, which considerably anticipates detection of AD first signs.

## Introduction

The genetic bases of behavior have long been an exclusive matter of study for evolutionary biologists intended to verify the inheritance and conservation of behavioral traits across generations or species (Atchley and Fitch, 1993). The advent of behavioral genetics in the first decade of the 20th century represented the first attempt to estimate the weight of genomic variations in the expression of behavioral phenotypes (Dobzhansky, 1937; Fuller and Thompson, 1960). In this context, the utilization of tools specific to classical genetics in rodent populations brought to light three fundamental principles. First, complex behaviors are influenced by a very large number of genes, but the individual effect of each gene is very small. Second, complex behavioral traits cannot be classified into discrete categories but are continuously distributed in a way that approximates a normal (Gaussian) curve, with the majority of individuals around the central values of the distribution exhibiting similar phenotypes. Third, artificial selection methods (bidirectional selection or inbreeding) allow to generate subpopulations showing well-differentiated, sometimes opposing, phenotypes, whereas individuals in each subpopulation exhibit a remarkable phenotypic homogeneity. These observations highly attracted the attention of neuroscientists who identified a powerful tool in these methods to investigate variations in neural substrates underlying extreme, although normal, variations in behavior.

Corollary to the demonstration that gene control behavior is the assumption that gene dysfunctions are pathogenic for behavior. In the late 20th century, molecular genetics made it possible to identify mutated genes in human patients showing a variety of diseases including those impacting cognition. The possibility to insert those genes in the mouse genome led to build up model organisms expected to recapitulate disease-specific neural and behavioral hallmarks. This objective was, however, partially achieved. Restricting our survey of the literature to data from Alzheimer's disease (AD) mouse models, it is apparent that the multiplicity of genomic manipulations aimed at overexpressing the three main human mutated proteins (APP, Tau, and presenilin (1) separately or jointly in likewise multiple wild-type (Wt) genetic backgrounds did not entirely reproduce, or even failed to model, the symptoms of human pathology. Hence, strategies to refine the face validity of AD models have been established, with a majority of those consisting in producing mutations closer to those observed in patients, and with little consideration of the cognitive profile of Wt mice, these mutations were expected to disrupt. Because neurodegenerative processes in AD patients start to develop in the temporal lobe, disrupt episodic and spatial memory but preserve motor-based procedural memory (Eldridge et al., 2002), the aim of this short review is to outline the importance of including (i) the cognitive specialization of inbred mouse strains used as recipient backgrounds and (ii) the choice of protocols anticipating detection of AD first signs, among the criteria to refine the face validity of AD mouse models.

### What Is an Inbred Mouse?

#### Inbreeding

Inbreeding consists of mating closely related individuals (sisters and brothers) taken from random bred populations for about 20 generations to produce a subpopulation whose members are homozygous, that is, have the same genotype. Inbred mice were initially generated by physiopathologists who identified the advantage of having individuals ruling out genetic variance and showing homogeneous traits to better circumscribe the nature and inheritance of several pathologies. For example, criteria of selection to start the production of inbred lines were “predisposition to develop neoplasia” to determine if cancer was inherited (Little, 1915), or “immunohistocompatibility response” to estimate the best immunological markers for tissue transplantation (Hellstrom, 1963). Incidentally, Bagg (1920) tested various inbred strains of mice in several multiple-choice mazes and found that the learning performance strongly varied between strains, whereas it was remarkably homogeneous within each strain. Later on, Vicari (1929) compared the time spent by DBA/2 and BALBc/ inbred mice, as well as by “Japanese Walzer” and “myencephalic bleb” mutant mice, to run a three-unit maze and observed that maze running times were strain-dependent. Some decades later, two seminal studies focusing on interstrain differences in learning and memory (Dennenberg, 1959; Bovet et al., 1969) have established the bases for behavioral genetics. Clearly, one advantage of inbreeding for neuroscientist interested in the genetic control of learning abilities is that no learning criterion is involved in the selection process. Thus, possible confounders like those evoked in the case of bidirectional selection where, for example, low and high learners in active avoidance might simply those having low and high pain thresholds or being less or more anxious (Río-Ȧlamos et al., 2015), were excluded. Another issue to be considered is that if inbred individuals are like homozygous twins, they all exhibit the phenotype of one single random bred individual. This means that their behavioral and neural traits are not distributed according to a normal curve, so an inbred population is in no way representative of a natural outbred population. As many strains have been accurately characterized, any neuroscientist interested in analyzing a particular behavioral or neural phenotype can select *a priori* either one strain expressing the behavioral or neural trait of interest, or several strains to be compared for their difference relating to this specific trait. Among the most commonly used strains, pure inbred (C57BL/6J, DBA/2J, BALB/c, FVB/NJ, 129SvEvTac) or mixed backgrounds (B6J/SJLJ) are predominant (Sultana et al., 2019). The DBA/2J mouse strain is often used to contrast the C57BL/6J strain, given that their genotype (Bottomly et al., 2011) and phenotype (Ingram and Corfman, 1980) are opposed in several aspects.

#### Inbred Mice Show Task-Specific Learning and Memory Performance

At the dawn of neuroscience, the most popular tasks performed to investigate learning in rodents were those designed by experimental animal behaviorists, which prevalently required to form motor habits or stimulus–response associations, and in which C57BL/6J mice (C57) were identified as poor learners. Specifically, C57 performed worse than BALBc, or DBA/2J (DBA), in the Lashley maze (Oliverio et al., 1972), the active avoidance (Bovet et al., 1969; Oliverio et al., 1972; Weinberger et al., 1992), and in situations of operant or instrumental conditioning (Renzi and Sansone, 1971) in which an elemental stimulus was used to initiate or stop responding. When O'Keefe and Nadel (1978) and Olton et al. (1978) identified the neural basis of spatial cognition in the hippocampal place cells, spatial tasks like the radial arm maze and the water maze were the golden standards to investigate cognitive functions and their alterations in rodents, especially in view of data showing that Alzheimer's disease (AD) patients with temporal lobe neurodegeneration were selectively impaired in episodic/declarative/spatial memory. We started testing C57 and DBA mice in a radial maze (Ammassari-Teule and Caprioli, 1985) and observed that C57 outperformed DBA, thereby reverting their previous status of bad learners. These findings were confirmed in other spatial protocols (Upchurch and Wehner, 1988, 1989; Passino et al., 2002) and extended to tasks which strongly rely on the hippocampus like reactivity to spatial novelty (Thinus-Blanc et al., 1996), contextual fear conditioning (Stiedl et al., 1999; Ammassari-Teule et al., 2000; Restivo et al., 2002), cross-maze place learning (Middei et al., 2004), and, very recently, pattern separation (Dickson and Mittleman, 2022). The C57 predisposition to do well in hippocampus-dependent tasks then prompted several groups to examine the structural and functional properties of their hippocampus. Compared to other strains, C57 were found to show a higher density of mossy fibers in the region inferior to the hippocampus (Barber et al., 1974; Lipp et al., 1988; Schwegler et al., 1988), an increased activity of hippocampal, but not cortical, protein kinase C (Wehner et al., 1990), and long-term stronger hippocampal potentiation (Matsuyama et al., 1997; Nguyen et al., 2000; Gerlai, 2001a; Jones et al., 2001).

Considering strain-specific levels of performance, it became rapidly evident that a majority of tasks in which C57 performed poorly were those in which DBA performed well, and vice versa. Beyond their aforementioned superiority in the Lashley maze and active avoidance, DBA mice were found to score better than C57 in cue-based fear conditioning (Paylor et al., 1994; Ammassari-Teule et al., 2000) and ethanol-induced conditioned place preference (Cunningham and Shields, 2018). On the one hand, these findings pointed out the remarkable ability of DBA to detect relevant elemental sensory stimuli to rapidly form stimulus–response associations in pavlovian or instrumental conditioning paradigms, or to implement egocentric orientation in spatial tasks. On the other hand, their inability to form configural representations, either of the aversive context in conditioning tasks or of distal environmental cues for allocentric orientation in spatial tasks, was interpreted as the consequence of the poorly functional morphological, biochemical, and plastic properties of their hippocampus, which led to consider them as a natural model of hippocampal dysfunction (Paylor et al., 1993). Supporting this view, DBA mice behave randomly in the hippocampus-dependent simultaneous olfactory discrimination task and do not form training-induced dendritic spines in the hippocampus (Restivo et al., 2006).

#### To Have, or Not to Have, an Outstandingly Functional Hippocampus: Advantages and Limitations in Relation to Different Memory Systems

The notion of memory systems arises from observations initially carried out in human subjects (Cohen and Squire, 1980) and later in rodents (Packard et al., 1989; Packard and White, 1991; McDonald and White, 1993) that different types of memory are governed by dissociable brain substrates. For example, episodic, declarative, spatial, or context-based memory are supported by temporal lobe regions, among which the hippocampus plays a central role, whereas stimulus–response associations and procedural memory, including motor habits, are overall controlled by the striatum. The independence of memory systems was demonstrated by data showing that disrupting the neural support of one system leaves unaltered the operations supported by the other system or even improves the preserved system by suppressing conflictual responding (McDonald and White, 1995).

In a majority of individuals, memory systems can be activated separately, concurrently, or sequentially, depending on the situation to copy with. For example, in the plus maze task (Packard and McGaugh, 1996), rodents were first trained to turn left to find a food reward in the unique baited arm and then exposed to probe trials in which they were released from the opposite starting arm. In those trials, they could either reproduce the motor response reinforced during training (turning left) and do not go to the baited arm or invert it (turning right) and go to the baited arm. Interestingly, the rats were found to turn right after a short training duration (1 week), consistent with hippocampus-based place learning, but to turn left after long training duration (3 weeks), consistent with striatum-based response learning. Interestingly, inactivation of the hippocampus after short training made the rats show striatum-based motor learning, whereas inactivation of the dorsolateral striatum after long training made them show hippocampal-dependent place learning. The point is that when C57 and DBA were trained in the same plus maze task, C57 showed place learning and predominant hippocampal activation after both short and long training, whereas DBA never relied on a particular system even they prevalently activated the dorsolateral striatum (Passino et al., 2002). Indeed, the consequence of the C57 inability to disengage the hippocampus is that inactivation of this region at any probe trial indeed disrupts place learning but does not promote response learning. Similarly, the consequence of the DBA/2 inability to rely on a particular system is that the inactivation of any region at any probe trial does not promote neither place nor response learning (Middei et al., 2004). Thus, contrary to what happens in rats, disrupting the neural substrate of one memory system but does not promote the utilization of another system in these genotypes. Indeed, the C57 propensity to engage the hippocampus in any situation they face is also observed in fear conditioning (FC) paradigms. Specifically, studies dissecting the neural bases of FC in outbred populations of rats have identified (i) tone fear conditioning (TFC) as an elemental associative learning system involving the basolateral amygdala (BLA) but not the dorsal hippocampus (Phillips and LeDoux, 1992; Paré et al., 2004) and (ii) contextual fear conditioning (CFC) as a configural learning system involving by both regions (Selden et al., 1991; Phillips and LeDoux, 1992). Different from that observed in these populations, C57 mice were found to concurrently activate the BLA and the dorsal hippocampus in both CTC and CFC, although they showed considerably less freezing in TFC than in CFC (Pignataro et al., 2013). In line with the view that recruiting the hippocampus in TFC is an obstacle to implement elemental stimulus–response associations, lesions to the dorsal hippocampus were found to enhance C57 TFC performance (Ammassari-Teule et al., 2002).

#### Modifications of Experimental Parameters or Rearing Conditions Can Abolish Interstrain Differences in Learning and Memory

The aforementioned experiments show that C57 predominantly form configural environmental representations in which elemental stimuli are embedded in and need to be disentangled to predict reinforcement and guide behavior. Thus, any manipulation of experimental factors that facilitates disentangling is expected to enhance cue-based performance in this mouse strain. This possibility was demonstrated in a study in which C57 and seven other mouse strains including DBA were trained to press a lever upon presentation of an elemental stimulus (tone or light) to avoid delivery of an electric footshock. DBA showed superior avoidance performance when the tone or the light was of short duration. However, a gradual increase in the stimulus duration was found to progressively abolish interstrain differences, thereby suggesting that C57 benefited for longer cue presentation to disentangle them from the context (Renzi and Sansone, 1971). Interestingly, Cunningham and Shields (2018) explored more recently the possibility that the most robust ethanol-induced conditioned place preference (CCP) shown by DBA compared to C57 might depend on strain differences in sensitivity to contextual cues. They found that compared to single cueing, multiple cueing increased CCP in both strains but that this effect disappeared more rapidly in DBA and was not sufficient to elevate the CCP performance of C57 to the level of DBA. They therefore concluded that CPP differences were due to a genotype-specific sensitivity to ethanol reward. Nevertheless, the fact that DBA mice outperform in amygdala-dependent conditioned taste aversion, that is, a task based on the association of a single gustatory stimulus with illness (Dudek and Fuller, 1978; Risinger and Cunningham, 1995), suggests a more global interpretation that DBA perform better due to the stronger BLA modulation of striatal-based elemental cue processing (Desmedt et al., 1998; Goode et al., 2016). Other examples which show that interstrain differences in fear conditioning vary or persist depending on whether freezing is recorded shortly or long after training (Nie and Abel, 2001; Balogh and Wehner, 2003) indicate that if the predominance of a memory system determines the nature of information each strain preferentially relies on, the time necessary to consolidate this information is a much important variable in the modulation of the behavioral responses. Indeed, manipulations like environmental enrichment (EE) or physical exercise modify cognitive abilities, attention, and anxiety in inbred mice but paradoxically exert improving or disrupting effects depending on the duration of and the age mice were exposed to these manipulations (Singhal et al., 2019). Among the principally observed strain-specific effects, EE was found to increase attention in C57 (van de Weerd et al., 2004), to either decrease (Chapillon et al., 1999) or increase (van de Weerd et al., 2004) anxiety in BALB/c, to reduce reactivity to novelty in both C57 and DBA (Dickson and Mittleman, 2021), and to accentuate C57 vs 129S6/SvEV differences in locomotor activity, anxiety, and social interactions (Abramov et al., 2008). Thus, no univocal, strain-independent beneficial effect of EE of behavior was observed.

## Alzheimer-Associated Mutations and Genotype of the Background Mouse

From the creation of the first transgenic mice, it clearly appeared that controlling the characteristics of the recipient strain was crucial to reveal or maintain expected transgene effects because inserting a mutated gene in different mouse strains was found to produce variable phenotypes and because it was observed in some cases that a phenotype was progressively losing its specificity due to non-specific mutations or uncontrolled environmental effects, thereby preventing data reproducibility. Multiple research groups with an expertise in mouse behavior genetics (Crusio, 1996; Gerlai, 1996, 2001b; Wehner and Silva, 1996; Crawley et al., 1997; Wolfer and Lipp, 2000; Lassalle et al., 2008) identified such risks and proposed several solutions.

### The Same Mutated Gene in Different Backgrounds Produces Different Phenotypes

#### First-Generation Models: Mutant HAPP Overexpression

One of the first transgenic murine models of Alzheimer's disease is the Tg2576 mouse developed by Hsiao et al. (1996) which overexpresses human APP (isoform 695) containing the double mutation K670N, M671L (Swedish mutation) under the control of the hamster prion protein promoter. This hemizygous mutation was originally introduced in a C57(B6) × SJL F1 hybrid background and stabilized by repeatedly backcrossing mutant mice with B6 × SJL F1 hybrids. The main issue addressed at that time was confounding effects due to the insertion of the mutated gene and those due to the transgene *per se*. It was therefore proposed to backcross the transgenic lines to one or even more inbred strains for at least three generations before performing phenotypic characterization in F1 hybrids. This strategy, however, revealed to be inadequate as repeated backcrossing to inbred lines produced non-specific performance impairments, reduced fertility, and, in some cases (FVB/N or B6), was found to be lethal. This prompted Lassalle et al. (2008) to insert the HuAPP695-SWE transgene in three different backgrounds (homogeneous: C57; heterogeneous: CBAJ; and hybrid: B6J/SJLJ) and to perform phenotyping in F1 generations after only one generation backcrossing. Comparisons included evaluation of anxiety in the elevated plus maze, spatial learning in the water maze, and fear conditioning. The results showed that Tg (+) C57 mice were globally more active and less anxious than their Tg (-) counterparts, as well as than Tg (+) and Tg (-) in other backgrounds. The calculation of a spatial index in the water maze revealed that even though this index was rather comparable between Tg (-) B6/SJL, C57, and CBA, the genetic background significantly modulated the expression of the transgene, with the lower spatial index being found when the mutation was expressed in the C57 background. Fear conditioning data did not provide evidence of differences in CFC performance between the three Tg (+) and Tg (-) backgrounds possibly because the experiments were carried out in 17-month-old female mice and, hence, recruited additive effects of sex and age to those of background and mutation. Nevertheless, this study provided the first demonstration that the behavioral characteristic of the recipient mouse was defining the degree of mutation-induced cognitive impairment. After two years, Rustay et al. (2010) compared the effect of the Swe mutation in the B6/SJL and 129 backgrounds and reported more deleterious effects at late ages in the 129 backgrounds for parameters that were not properly cognitive (locomotor activity, spontaneous alternation) and for which Tg (-) 129 were scoring lower than Tg (-) B6/SJL. Interestingly, models in which three (APP, PS1, and tau) or five (two APP and three PS1) familiar AD mutations were concurrently inserted in mouse genomes were predominantly developed in a C57 background (Sterniczuk et al., 2010; Forner et al., 2021; Sil et al., 2022) or hybrid backgrounds with a B6 component. Confirming the inadequacy of the DBA background for AD mutations, insertion of the APPswe and PSEN1de9 mutated genes in DBA exacerbated lethal seizures and even lessened amyloid deposition (Jackson et al., 2015).

#### Second- and Third-Generation Models: App Gene KI Mutations

These models were generated using the *App* gene knock-in strategy to overproduce pathogenic Aβ without overexpressing APP with the objective to avoid artifacts due to APP overexpression *per se*. Murine Aβ sequences (Swedish, Beyreuther/Iberian, artic) were humanized by changing amino acids that differ between mice and humans and then introduced separately or concurrently to generate APP^NL^, APP^NL−F^, or APP^NL−G−F^ mice expressing Wt human Aβ under control of the mouse App locus (Nilsson et al., 2014; Saito et al., 2014). The point is that if humanized Aβ does increase the face validity of these models as far as amyloidosis is concerned, the consequences of App-KI manipulation on neural and behavioral parameters do not differ much from those observed in the first-generation transgenic models. App-KI mice show the same increased glutamate release probability and intense astrocytosis/gliosis around the Aβ plaques, discordant results regarding hippocampal LTP (decreased or intact), and late memory impairments (Nilsson et al., 2014; Baglietto-Vargas et al., 2021; Benitez et al., 2021). Furthermore, the fact that robust amyloidosis and its metabolic consequences took at least 18 months to emerge has led to the development of third-generation models, that is, double KI mutants obtained by crossing APP^NL−G−F^ mice with mice bearing PS1-KI (Sato et al., 2021) to accelerate the detection of mutation effects. In these studies, however, (i) the genetic background (until now C57) is never mentioned in the method section of articles, suggesting that it is *per se* irrelevant if the mutant mouse is viable; (ii) the focus is predominantly placed on the pathogenic inflammatory/metabolic cell alterations engendered by multiple Aβ species and on the possibility to rescue them by rectifying the mutated genomic sequences; (iii) mutation effects are investigated separately at the neural (Jun et al., 2020) or behavioral (Sakakibara et al., 2019; Sutoko et al., 2021) levels and therefore do not inform on the status of neural circuits when mice face cognitive challenges, that is, when these circuits actually come into play.

## Systems Neuroscience Approaches

Memory formation requires changes in neuronal network connectivity mediated by modifications in the strength and number of synapses. Since the discovery that synapses are primary targets of Aβ oligomers (Selkoe, 2002), central to the validation, an AD mouse model is the demonstration that deficits in hippocampal-dependent memory associate with hippocampal synaptic dysfunctions. A survey of the literature indicates that dysfunctions including dendritic spine loss or long-term potentiation (LTP) deficit have been identified in the majority of AD mouse models but mostly under naive conditions. Increasing evidence reveals, however, that studying structural, functional, and molecular alterations which develop in the hippocampus when animals are given memory tasks anticipates detection of synaptic failure and unveils pathogenic or compensatory reorganization of brain circuits, which might otherwise not be observed in naive conditions.

### Training Discloses Neural Alterations in APP Mutants

Heterozygous B6-Tg/Thy1APP23Sdz (APP23) mice show amyloid plaques in the hippocampus (Sturchler-Pierrat and Staufenbiel, 2000) and severe deficits in hippocampal-dependent tasks (Lalonde et al., 2002; Vloeberghs et al., 2006) around 12 months of age. When trained in a water maze at the age of 7 months, they swim regularly but show increased latencies and travel a longer distance to find the submerged platform than Wt C57 controls. Nevertheless, the fact that both groups reach the same level of performance at the end of training indicates that mutant mice exhibit more delay in learning than mice with an incapability to learn. Following training, mice were euthanized to evaluate the effect of the learning experience on dendritic spines and synaptic activity. Spine density measured on CA1 neuron dendrites in non-training and pseudo-training conditions was not found to vary between mutant and Wt mice. Differently, more spines were counted post-training in the mutant mice, thereby indicating that circuits unaltered at rest undergo stronger learning-induced remodeling. Indexes of basal synaptic transmission like input–output curves and paired-pulse facilitation were indistinguishable between genotypes in all experimental conditions, but CA3–CA1 long-term potentiation decayed more rapidly in the mutant mice (Middei et al., 2010). Together, these findings allow the following conclusions to be made. The observation that mutant mice perform same as the Wt mice at the end of training suggests that formation of novel synapses might compensate for the rapid decay of synaptic plasticity.

### Synaptic Compensatory Mechanisms in 2-Month-Old 3xTg-AD Mice

The 6–8-week-old 3xTg-AD mice exhibit intact synaptic plasticity at rest. Nevertheless, differently from Wt mice, they show increased synaptic depression when their synaptic homeostasis is altered by suppression of ryanodine receptor (RyR)-evoked calcium signaling. The authors hypothesize that in baseline conditions, 3xTg-AD mice exhibit increased activity of this receptor which, by augmenting RyR-evoked calcium release, blocks the predisposition of mutant synapses to exhibit long-term depression (Chakroborty et al., 2012). The authors successively demonstrate that compensatory maintenance of synaptic plasticity is mediated by an augmentation of nitric oxide levels, a presynaptic regulator of calcium release which increases glutamatergic transmission (Chakroborty et al., 2015). Although this study does not examine the functional consequences of Ry-R manipulations *in vivo*, these data support the relevance of detecting compensatory synaptic changes in presymptomatic AD mice to be targeted by pharmacological approaches aimed to prolong them over time.

### Training Experience Reveals Neural Compensation in Pre-Symptomatic APP Mutants

Tg2576 mice and their Wt C57 controls trained for CFC at the age of 2 months show the same reactivity to footshocks and exhibit immediate c-fos activation in the dorsal CA1 region of the hippocampus and the basolateral region of the amygdala (BLA). When returned 24 h later to the safe training context, all mice show intense freezing, but differently from Wt mice, mutant mice do not exhibit any sign of c-fos activation or dendritic spine remodeling in CA1, instead they show c-fos overactivation and dendritic spine remodeling in BLA, in line with the view that the latter region compensates for hippocampus failure and sustains their intact CFC performance. Examination of Aβ levels 24 h after CFC in the mutant mice non-returned to the conditioning cage indicates a selective increase in Aβ42 oligomers in CA1 but not BLA. This is shown by Western blot analyses using the amino-terminal specific anti-Aβ42 antibody AD54D2 and the carboxy-terminal specific anti-Aβ42 antibody (clone 295F2), as well as by immunofluorescent detection of Aβ using the D54D2 and the carboxy-terminal specific antibody 12F4. In the Wt mice, the Aβ42 signal is about undetectable in both regions at rest, and no rise is observed following CFC. Thus, CFC learning triggers immediate release of Aβ species in the hippocampus of cognitively asymptomatic Tg2576 mutants (Pignataro et al., 2019). Validation of a causal link between the CFC-induced Aβ rise and absence of hippocampus activation/remodeling in cognitively asymptomatic Tg2576 mice comes from data which show that CFC-trained mutant mice receiving intra-hippocampus injections of DAPT, a gamma secretase inhibitor which reduced Aβ levels, show regular formation of hippocampal spines with no longer compensatory formation of spines the BLA. Therefore, this is the first study to provide evidence of neural compensation consisting of enhanced synaptic activity in brain regions spared by Aβ load. Furthermore, it unravels an activity-mediated mechanism by which neuronal activation produced during CFC encoding triggers Aβ oligomerization in the hippocampus and prevents synaptic rearrangements in this region. Indeed, the observation that learning activates compensatory circuits allowing mutant mice to maintain an intact memory delineates entirely novel therapeutic avenues in the AD field. Considering that “compensatory circuits” recruit regions unaffected by Aβ load, their stimulation might be more beneficial to prolong cognitive efficiency than stimulation of disrupted “canonical circuits.”

## Conclusion

### Is There an Ideal Background for Overexpressing Mutant APP?

Although it may appear trivial, the first requirement for the recipient background of an APP mutation is to exhibit sufficiently elevated episodic memory capacities likely to be significantly altered by the mutation. At the first sight, B6 mice appear appropriate given their optimal episodic memory scores, even though their outstandingly functional hippocampus is *a priori* not representative of the natural genetic heterogeneity of AD patients and even represents an obstacle to the natural evolution toward procedural memory (Passino et al., 2002) which becomes rapidly predominant in AD patients (Eldridge et al., 2002) and AD rat models (Ammassari-Teule et al., 2002). This limitation, however, is not fully overcome by inserting mutations in mixed backgrounds. For example, expression of APPswe in B6/SJL mice generates five mutant phenotypes (black, white-belly agouti, albino, tan with pink eyes, and silver with pink eyes), with the three later ones showing poor contextual memory due to vision problem. These observations highlight the importance of controlling the sensory phenotype of strains and substrains of mice, which can result in the loss of function (deafness, Zheng et al., 1999; blindness, Brown and Wong, 2007), as well as a gain of function (resistance to noise-induced hearing loss, Street et al., 2014; enhancement of olfactory conditioning in mice with vision defects, Brown and Wong, 2007).

The second requirement is to choose a strain showing a cognitive, even mild, deficit at a sufficient early age to have the performance of the wild-type counterpart unaffected by aging. Apparently, C57 mice align again with this criterion. For example, Tg2576 in a C57 background shows a CFC deficit associated with a decrease in hippocampal spines (D'Amelio et al., 2011) and the presence of Aβ oligomers already at 3 months of age (Pignataro et al., 2019), whereas the Tg2576 mutation in a B6/SJL background is still considered as a late AD model.

In addition, these observations raise another equally important question, namely, the choice of experimental protocols allowing to anticipate neural dysfunctions at a stage where no, or mild, cognitive impairment is observed to start therapies when maximal effectiveness can be expected.

### Alternative Strategies: Incorporating Genetic Diversity Into Mouse Models of AD

The fact that inbred mouse strains do not reflect the phenotypic variability observed in natural populations limits *a priori* the face validity of AD models. One strategy alternative to the insertion of human-like mutations in a single genome is increasing the genetic diversity of recipient mouse backgrounds to generate well-differentiated phenotypes carrying the same mutation. For example, Onos et al. (2019) B6 mice expressing APPswe and PS1de9 (APP/PS1) transgenes were backcrossed for six generations with three wild-derived strains (CAST/EiJ, WSB/EiJ, and PWK/PhJ). As expected, they obtained large phenotypic differences between mice substrains as far as cognitive ability, neurodegeneration, plaque load, cerebrovascular health, and cerebral amyloid angiopathy were concerned. Transcriptional analyses revealed, however, that the “strain” factor was the largest source of variation suggesting a potential risk of this breeding method, that is, the possibility that wild-type genomes include AD risk genes. For example, crossing B6 males with deletion of Cacna1c and Tcf7l2 genes associated with multiple psychiatric diseases with wild-type females from 30 inbred laboratory strains resulted in highly variable, sometimes opposing, effects (Sittig et al., 2016). Thus, if the translatability of data obtained by studying mutations in one single inbred genotype is limited, highly diversified backgrounds require careful genotypic/phenotypic characterization prior to insertion of mutations. With the same objective of differentiating AD phenotypes, the utilization of recombinant inbred (RI) strains overcame issues relating to unknown wild-type-derived genomes since they were established to detect gene segregation and linkage and for identifying associations between behavior and quantitative trait loci (QTL) accounting for variations in behavior (Plomin et al., 1994). RI strains were obtained by crossing two inbred parental strains (e.g., B6JxDJ) giving raise to F1 or F2 generations maintained under a strict inbreeding regimen. After at least 20 generations, individuals in each RI lines were found to be genetically homogeneous, whereas families of RI lines exhibited genetic diversity. Mapping of quantitative trait loci with small or very large effects allowed to build up genetic reference panels providing databases on genotypes x phenotype interactions (Peirce et al., 2004). Taking advantage of these databases, Neuner et al. (2019) created the AD-BXD panel of transgenic mouse strains. This panel was established by crossing 5xFAD female mice to male mice from the BJxDJ genetic reference panel until 27 F1 AD-BxD strains were generated. The 5xFAD mutation being hemizygous, half of mice carried no transgene and served as isogenic control for mutations. This method considerably extended AD phenotypes for traits including “age onset of symptoms” and “acceleration of memory decline” but also allowed to identify variations in genes regulation. Specifically, the expression of genes controlling neural activity, structure, and function was decreased, whereas the expression of immune response genes was increased. This approach appears undeniably insightful for personalized medicine if followed by systems neuroscience analyses estimating structure–function relationships in the subset of strains of interest for the trait under examination (e.g., early vs. late AD onset, abrupt vs. progressive emergence of cognitive alterations).

## Author Contributions

The author confirms being the sole contributor of this work and has approved it for publication.

## Funding

This work was funded by the Italian Ministry of Health (5XMille–2018).

## Conflict of Interest

The author declares that the research was conducted in the absence of any commercial or financial relationships that could be construed as a potential conflict of interest.

## Publisher's Note

All claims expressed in this article are solely those of the authors and do not necessarily represent those of their affiliated organizations, or those of the publisher, the editors and the reviewers. Any product that may be evaluated in this article, or claim that may be made by its manufacturer, is not guaranteed or endorsed by the publisher.
